# A Viral Labelling Study of Spinal Trigeminal Nucleus Caudalis Projection Neurons Targeting the Parabrachial Nucleus

**DOI:** 10.1111/jnc.70028

**Published:** 2025-03-06

**Authors:** Sophie Maric, Mehedi Hasan, Madison L. Pounder, Brett A. Graham, Tyler J. Browne

**Affiliations:** ^1^ School of Biomedical Sciences & Pharmacy, Faculty of Health University of Newcastle Callaghan Australia; ^2^ Hunter Medical Research Institute (HMRI) New Lambton Heights Newcastle New South Wales Australia

## Abstract

Projection neurons (PNs) in the Spinal Trigeminal Nucleus Caudalis (Sp5C) relay orofacial nociceptive information to higher brain regions such as the thalamus and the parabrachial nucleus (PBN). Our understanding of Sp5C PN organisation and function has advanced less than the parallel spinal cord output system despite their corresponding roles for transmission of nociceptive signals from the orofacial region and body respectively. Viral vectors are an established approach for studying circuit connectivity in the nervous system, but different serotypes are known to produce variable results across circuits. As such, we sought to validate the utility of two common viral serotypes in spinal PN research: retrograde adeno‐associated virus serotype 2 (rgAAV) and adeno‐associated virus serotype 9 (AAV9), for identifying and analysing Sp5C PNs that project to the PBN. Following unilateral injections of either viral serotype into the PBN, many Sp5C projection neurons were retrogradely labelled. For both serotypes, these injections labelled Sp5C PNs bilaterally with a strong bias to the ipsilateral Sp5C. Within Sp5C, similar levels of PN labelling were present in both superficial and deep regions, contrasting previous work in spinal PNs that showed greater labelling by AAV9 versus rgAAV. Comparisons of the age dependence of labelling showed greater retrograde labelling of Sp5C projection neurons when injections were made in young adult animals. Finally, we demonstrate successful Cre‐dependent recombination to selectively express channelrhodopsin‐2 in Sp5C projection neurons. Together, these experiments show that rgAAV and AAV9 produce strong Sp5C PN transduction and provide a basis for future study of the afferent and efferent functions of the Sp5C PN population in health and disease.

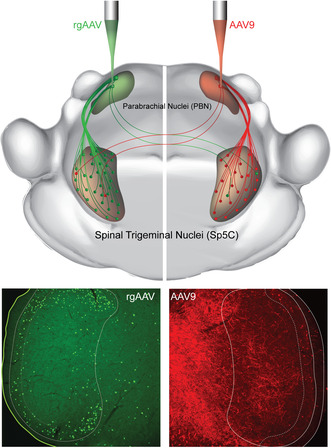

## Graphical abstract

Using adeno‐associated viral vectors (AAVs) to label projection neurons (PN) that link nervous system regions is a powerful tool in neuroscience for studying circuitry and function. However, AAVs exhibit tropism, which can produce variable expression depending on the viral serotype and the CNS region targeted. The cartoon summarises our comparison of two AAV serotype and promoter combinations to label PNs of the orofacial region of the ascending sensory system, linking the spinal trigeminal nucleus caudalis (Sp5C) to the parabrachial nucleus (PBN). This targeting strategy employed retrograde AAV (rgAAV; below; left) and AAV9 (below; right), and yielded robust PN labelling bilaterally within Sp5C, with a strong bias to the ipsilateral (injection) side, as depicted in the photomicrographs.

**Abbreviations**
AAV9: Adeno‐associated Viral Vector Serotype 9
Cre: Cre Recombinase
eGFP: enhanced Green Fluorescent Protein
LI‐LV: Rexed Laminae 1–5
MDH: Medullary Dorsal Horn
PBN: Parabrachial Nucleus
PN: Projection Neuron
RFP: Red Fluorescent Protein
rgAAV: Adeno‐associated Viral Vector Retrograde serotype
Sp5C: Spinal Trigeminal Nucleus Caudalis

## 1 Introduction

The orofacial region receives extensive sensory innervation, reflecting the central role that the head and face have on an organism's social and physiological health (Gambeta, Chichorro, and Zamponi 2020). Furthermore, there are unique components of orofacial innervation such as the convergence of innervation from multiple cranial nerves and upper cervical nerves, centrally located proprioceptive sensory afferent neurons, an increased proportion of myelinated primary afferent fibres, and direct interaction with cerebellar arteries that contribute to both normal sensory perception and the progression to chronic pain (i.e., neurovascular compression of the trigeminal ganglia implicated in the development of hypersensitivity). Orofacial pain broadly arises from neurogenic, somatic, and psychogenic sources in facial, head and neck structures (Bičanić et al. 2019; Pertes and Heir 1991). This includes trigeminal neuralgia, headaches (cluster and tension types) (Stewart et al. 2003), migraines (Goadsby and Hargreaves 2008), dental and oral muscle or joint disorders (Li et al. 2024) and different head and neck pains (Romero‐Reyes and Uyanik 2014). Unfortunately, the underlying mechanisms and pathology in these conditions remain poorly understood, rendering orofacial pain difficult to diagnose and treat. This often leads to devastating personal and social impacts on an individual, who may completely withdraw from social and physical activities in an attempt to avoid triggering bouts of spontaneous pain (Türp and Gobetti 1996; Zakrzewska et al. 2017). Poorly treated chronic pain from the head and neck can also lead to time spent out of the workforce and engagement in daily life, underpinning a substantial economic burden (Stewart et al. 2003).

Further compounding the lack of effective management strategies for pain arising from the head and neck, a detailed understanding of normal sensory and pain processing within this region is lacking. In broad terms, the pain pathway for the orofacial region starts with the stimulation of nociceptors in the face, head or neck, with trigeminal nerve (V) afferents carrying this input to the spinal trigeminal nucleus caudalis (Sp5C), also referred to as the medullary dorsal horn (MDH) (Gambeta, Chichorro, and Zamponi 2020). Here, noxious signalling is relayed mainly to laminae I, II and V. Local circuit interneurons in these laminae can then modulate sensory information, before Sp5C output neurons relay processed signals to a range of targets, including the sensory thalamus (Li et al. 2019), the parabrachial nucleus (PBN) (Tokita et al. 2009), the caudal (Panneton, Gan, and Livergood 2011) and rostral ventrolateral medulla (de Preter and Heinricher 2023), as well as the periaqueductal grey (Chang, Okamoto, and Bereiter 2012). In this context, the Sp5C is the first central site to process noxious orofacial information, and parallels the spinal dorsal horn (DH) in its role for sensory information from the body (Dubner and Bennett 1983; Sessle 1987). Unlike the spinal DH, however, our understanding of the neuronal populations and circuits within the Sp5C has received less attention.

Regardless of whether sensory signals originate from the body or the head, face and neck region, the key role of relaying this processed sensory information to the brain is fulfilled by projection neurons (PNs). Within the spinal DH, PNs have been shown to represent a small fraction of the local neuronal population but exhibit distinct regional distributions and constitute a range of genetically and functionally distinct subtypes (Bell et al. 2024; Browne et al. 2021; Chen et al. 2024; Wercberger et al. 2021). Information on the function of these critical sensory pathways is rapidly expanding with ongoing advances in transgenic and viral techniques, enabling detailed and selective analysis of spinal DH projection populations (Chen et al. 2024; Choi et al. 2020). These approaches have yet to be fully applied to study the Sp5C PNs. Specifically, adeno‐associated viruses (AAVs) have been instrumental to recent advances in functional anatomy, targeted electrophysiology and optogenetics‐assisted circuit mapping, as well as understanding behavioural consequences of spinal and cortical circuit activity. Providing advantages over classic tracers, AAVs are able to comprehensively label entire neuronal pathways and resolve their postsynaptic targets and local organisation (Haenraets et al. 2017; Tervo et al. 2016) yielding the ability to specifically assess distinct subnuclei and microcircuits. A common challenge in these modern analysis techniques, however, is the unpredictable nature of transduction efficiency, relating to viral serotype and promoter actions in different cell populations and CNS regions (Aschauer, Kreuz, and Rumpel 2013; Burger et al. 2004; Haenraets et al. 2017; Issa et al. 2023).

In this study, we assess the utility of two AAV serotypes we have previously characterised in studies of spinal DH projection neurons (Browne et al. 2024; Browne et al. 2021) to label Sp5C PNs. By injecting AAVs into the PBN, a key efferent target of Sp5C (Cechetto, Standaert, and Saper 1985; Feil and Herbert 1995; Panneton and Burton 1985), we characterise the density and location of retrogradely labelled Sp5C projection neurons, a critical first step in confident, selective targeting of components of the trigeminal output pathway.

## 2 Methods

All surgical and experimental procedures were approved and undertaken in accordance with the University of Newcastle Animal Care and Ethics Committee (specifically Animal Research Authorities A2018‐828, and A2024‐406) and adhered to the ARRIVE guidelines (Kilkenny et al. 2010). Experiments used wild‐type C57BL/6 mice (sourced from Australian BioResources, Moss Vale), or the homozygous, Ai32 Cre‐dependent ChannelRhodopsin‐2 (ChR2) animal (JAX: 012569: Strain details: B6;129S‐Gt(ROSA)26Sortm32(CAG‐COP4 × H134R/EYFP)Hze/J (Madisen et al. 2012)). Mice were held in an animal care facility with *ad libitum* access to food and water and kept on a 12‐h light/dark cycle. Animals were group‐housed in open‐top cages, three per cage until injection surgeries were completed, at which time the animals were single‐housed in recovery until experimental endpoints. Neuroanatomical mapping of trigemino‐parabrachial (Sp5C‐PBN) neurons in mice was achieved by injection of either AAV2‐retro‐hSyn‐Cre‐eGFP (wild‐type: 9 weeks; *n* = 3 and Ai32: 23 weeks; *n* = 3) or AAV9‐Cb7‐Cl‐mCherry (age 26 weeks; *n* = 3) into the parabrachial nucleus (PBN). Sources, titres and specific virus details are presented in the key resources table (Table 1).

**TABLE 1:** Resources and reagents.

Virus class	Construct	Titre (GC/mL)	Source	Catalogue #	Acknowledgement
Fluorescent reporter	pENN.AAV.CB7.CI.mCherry .WPRE.RBG (AAV9‐RFP)	2.5 × 10^13^	Addgene	105 544‐AAV9	Gift by James M. Wilson
Cre recombinase + fluorescent reporter	pENN‐AAV‐hSyn‐HI‐eGFP‐Cre.WPRE.SV40 (rgAAV‐GFP)	1.9 × 10^13^	Addgene	105 540‐AAVrg	Gift by James M Wilson

Antibody	Details	Conc.	Source	Catalogue #	RRID
Primary	Chicken anti‐GFP	1:1000	Abcam	Ab13790	AB_2936447
Primary	Goat anti‐RFP	1:1000	Jomar life research	200–101‐379	AB_2744552
Primary	Mouse anti‐Cre	1:500	Millipore	MAB3120	AB_2085748
Secondary	Donkey Anti‐Mouse‐Cy3	1:500	Jackson immunoresearch	715–165‐150	AB_2340813
Secondary	Donkey Anti‐Chicken‐FITC	1:500	Jackson immunoresearch	703–095‐155	AB_2340356
Secondary	Donkey Anti‐Goat‐AF594	1:500	Jackson immunoresearch	705–585‐003	AB_2340432
Nissl stain	NeuroTrace Red (530/615)	1:100	ThermoFisher	#N21480	
Nissl stain	Neurotrace Green (500/525)	1:100	ThermoFisher	#N21482	

### 2.1 Intracranial Viral Injections

Mice underwent surgery for intracranial injection of the viral constructs AAV9‐Cb7‐Cl‐mCherry (AAV9‐RFP; *n* = 3) or AAV2‐retro‐hSyn‐Cre‐eGFP (rgAAV‐GFP; *n* = 6) into PBN. Briefly, mice were anaesthetised with isoflurane (5% induction, 1.5%–2% maintenance), secured in a stereotaxic frame (David Kopf Instruments, Model: 963/902, Tujunga, CA, USA), and body temperature maintained by feedback homeothermic blanket at 37°C (Thermostar Model: 69026, RWD Life Science Co., LTD, Shenzhen, Guangdong, China). Supportive analgesia (5 mg/kg carprofen subcutaneous) was administered at the beginning of surgeries. Craniotomies were made with a 0.5‐mm dental drill using the stereotaxic coordinates of 5.25 mm posterior to bregma, and −1.2 mm lateral to the midline, adapted from The Mouse Brain Atlas, ensuring that each animal received an injection to the right PBN (Paxinos 2001). An injection pipette was then advanced to a depth of 3.6 mm from the skull surface to administer a unilateral PBN injection of viral sample (total volume~200–300 nL) delivered via a Nanoject III Programmable Nanoliter Injector (Drummond Scientific Company, Broomall, PA, USA). Virus was injected at a rate of 1 nL/s, with a 60‐s pause at the midway point of the total volume. The pipette was initially left in place for 7 min after completion of injection before withdrawing 200 μm and allowing a further 2‐min rest time to minimise the virus sample being drawn back up the pipette track. Based on other retrograde labelling studies (Haenraets et al. 2017) and our own observations (Browne et al. 2024; Browne et al. 2021) we adopted a 10‐ to 14‐day period for viral permeation to allow sufficient projection neuron labelling before Sp5C sections were prepared. All animals were monitored daily for changes in exploratory behaviours, locomotion, weight, dehydration and received supportive analgesia (carprofen; 5 mg/kg subcutaneously) for the immediate recovery period (3 days). All animals included in this study made uneventful recoveries from surgery and showed no overt behaviours to indicate injection‐related pathology.

### 2.2 Tissue Processing and Immunolabelling

At the conclusion of the infection time following viral injection, animals were overdosed with sodium pentobarbital (200 mg/kg) and underwent rapid transcardial perfusion with saline (~5 mL; 0.9% w/v sodium chloride) followed immediately by 4% paraformaldehyde in 0.1 M phosphate buffer (> 24 mL). Brains were removed following decapitation at the superior margin of the rib cage to ensure that the trigeminal nucleus caudalis was left intact for subsequent processing.

**FIGURE 1:**
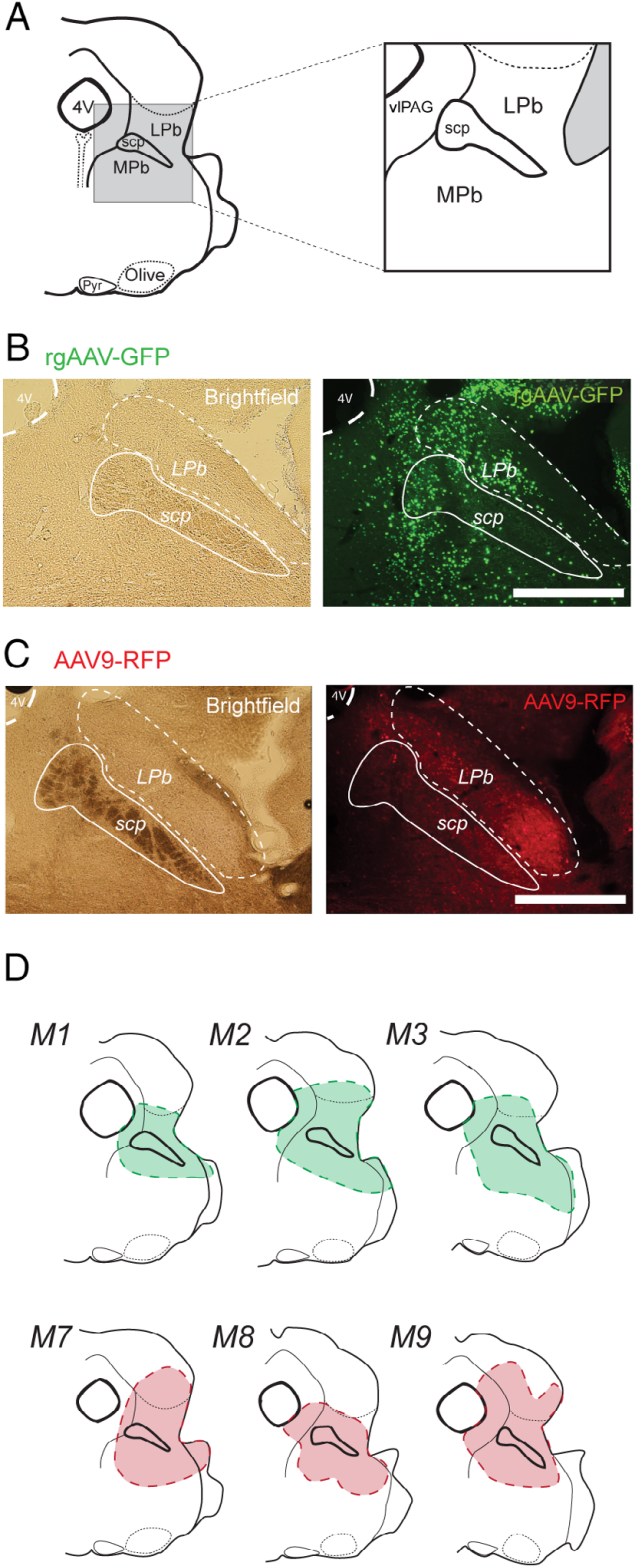
Parabrachial Nucleus viral injection sites. (A) Schematic shows key brainstem landmarks used to identify the PBN and its subnuclei in a coronal section at the level of the rostral pons, including the lateral parabrachial nucleus (LPb), medial parabrachial nucleus (MPb) and the superior cerebellar peduncle (scp) that separates them. Other notable landmarks include the pyramids (Pyr), olives (Olive) and the fourth ventricle (4 V). (B) Images show example rgAAV‐GFP injection site labelling. Brightfield image (left) shows landmarks for PBN location and the fluorescence image (right) shows rgAAV‐GFP labelling that is concentrated in the PBN. (C) Image shows example AAV9‐RFP injection site labelling. Brightfield image (left) confirms PBN location, and the fluorescent image (right) shows AAV9‐RFP labelling that is concentrated in the PBN. (D) Summary maps show injection sites across three mice highlighting the consistency of viral injections in the PBN for both rgAAV (top; green; M1, M2, M3) and AAV9 (bottom; red; M7, M8, M9). All injection site images were assessed using a 4× objective. Scale: B, C: 500 μm.

All brains were sectioned both at the level of the Spinal Trigeminal Nucleus Caudalis (Sp5C), to assess Sp5C‐PBN projection neuron labelling, and the PBN to verify inclusion of this structure with the viral injection site (Figure 1). Brains were first submerged in 0.1 M PBS with 30% sucrose (w/v), and once equilibrated, frozen and sectioned on a cryostat (50 μm; Leica CM1900). Following sectioning, wild‐type brain sections were washed in 0.1 M PBS and transferred into anti‐GFP or anti‐RFP; Ai32 brain sections were transferred into anti‐GFP and anti‐Cre recombinase primary antibodies (Table 1). All antibodies were made to final concentration in 0.3 M PBS with 0.3% Triton‐X and 10% normal donkey serum. After overnight incubation, sections were washed (3 × 15 min in 0.1 M PBS), transferred to species‐specific secondaries (Table 1) in 0.3 M PBS with 0.3% Triton‐X, and left overnight. Wild‐type sections were then washed as above before being immersed in Neurotrace fluorescent Nissl stain for 30 min, after which they were further washed. All brain sections were mounted on slides and covered in Fluorogel mounting media with DABCO fluorescence stabiliser (Proscitech, Thuringowa, QLD, Australia: IM037). Brain injection sites were imaged using a 1.25× objective to capture both brightfield and fluorescent images. Tissue was included in this study if fluorescent labelling was confirmed in the PBN. No animals were excluded from this study.

### 2.3 Sp5C Imaging and Neuronal Counting

For the analysis of Sp5C‐PBN projection neurons, sections were imaged using a Crest Optics X‐Light V3 spinning disk confocal microscope equipped with a 70‐μm numerical aperture and using a 10× air or 20× water objective (z‐step = 1 μm; field size = 1350 × 1350 and 675 × 675 μm respectively). Middle to caudal regions of Sp5C were imaged for analysis based on the presence of obvious landmarks, including the pyramidal decussation and central canal caudal to the obex. The boundaries of Sp5C were defined using previously described landmarks and structures (Gobel et al. 1977) and aided by the neuronal packing delineated with Neurotrace staining. Briefly, Sp5C resembles an elongated kidney shape encapsulated laterally by superficial white matter and incorporated substantia gelatinosa, a translucent section of grey matter that runs along the lateral aspect of brainstem sections in this region. We defined a deep (medial) Sp5C border at the point where the reticulated formations became prominent and there was a distinct change in neuronal size and packing. Cellular counting was restricted to within Sp5C and was undertaken manually. Cellular profiles were required to be discretely labelled and clearly discernible from the background fluorescence for inclusion in counting. They also exhibited neuronal‐like profiles (round, spheroid 3D structures), with definable edges, and appeared in multiple consecutive optical sections. Neuronal location data was analysed using an ROI‐based method within ImageJ (Schindelin et al. 2012). This assigned each GFP/RFP positive cell profile as an ROI, with corresponding X, Y coordinates positioned at the centre of the cell body. Counting was undertaken bilaterally, quantifying the number of GFP/RFP labelled profiles ipsilateral and contralateral to the PBN injection site across the same section. To assess the regional distribution of labelled Sp5C‐PBN projection neurons, the Sp5C was then divided into superficial and deep regions by adding a line that ran parallel and 100 μm medial to the lateral border of Sp5C. All Sp5C‐PBN projection neurons within this 100‐μm‐wide region (incorporating laminae I–II) were defined as superficial, and all cells within the remainder of Sp5C (incorporating laminae III–V) were defined as deep. All identified cell positions were also plotted in summary maps, using the assigned coordinates, allowing counts and distributions to be collapsed across sections and animals for group comparisons. Colocalisation of ChR2 expression and Cre expression was undertaken by first annotating all ChR2 profiles and then assessing for Cre colocalisation in the corresponding channel. Neurons were counted as colocalised if the Cre profile was in sequence with the ChR2 expression and completely within the ChR2‐eYFP labelled soma.

### 2.4 Statistical Analysis

All values presented are the mean ± standard deviation, and sample sizes were not calculated prior to analysis. Animal numbers were based on studies of a similar nature (Browne et al. 2021; Haenraets et al. 2017). Shapiro–Wilk tests for normality were used to inform our use of testing between labelled samples. Comparisons between the age of viral injection, viral serotype labelling, as well as between the superficial and deep Sp5C‐PBN neuron populations were performed using an independent samples *t*‐test. Where distributions did not meet the normality assumption, the Mann–Whitney U test for independent samples was utilised. Comparisons between animals that received the same virus were made using one‐way ANOVAs. Where assumptions of normality or homogeneity of variance were not met, a Kruskal–Wallis one‐way ANOVA or Welch's ANOVA was utilised respectively. Tukey's post hoc test was utilised to further determine differences between groups. Statistical significance was accepted as *p* < 0.05. All statistical analyses were performed using IBM SPSS Statistics v29.0.1.0. No test for outliers was performed.

## 3 Results

This study assessed the ability of viral constructs, increasingly used to study spinal cord projection populations (Browne et al. 2024; Chen et al. 2024; Choi et al. 2020), to label trigemino‐parabrachial (Sp5C‐PBN) projection neurons of the brainstem. Both serotypes identified Sp5C‐PBN projection neurons in superficial and deep Sp5C regions, though the distribution and number of each population varied depending on the viral construct and age. On average, the rgAAV‐GFP and AAV9‐RFP constructs labelled a similar number and distribution of Sp5C‐PBN projection neurons. This observation was also reflected in the number of Sp5C‐PBN projection neurons identified both ipsilateral and contralateral to the PBN injection site as well as within the superficial and deep populations. Interestingly, a comparison of rgAAV‐GFP labelling achieved when virus was injected in young versus older adult animals showed that greater expression was achieved in younger animals. Finally, injection of virus into a transgenic Cre‐dependent mouse line yielded strong, selective channelrhodopsin‐2 (ChR2) expression. Together, these observations confirm the suitability of these viral constructs to identify and study the output neurons within Sp5C that relay sensory information from orofacial areas to higher brain regions.

### 3.1 rgAAV Labelling of Sp5C‐PBN Projection Neurons

**FIGURE 2:**
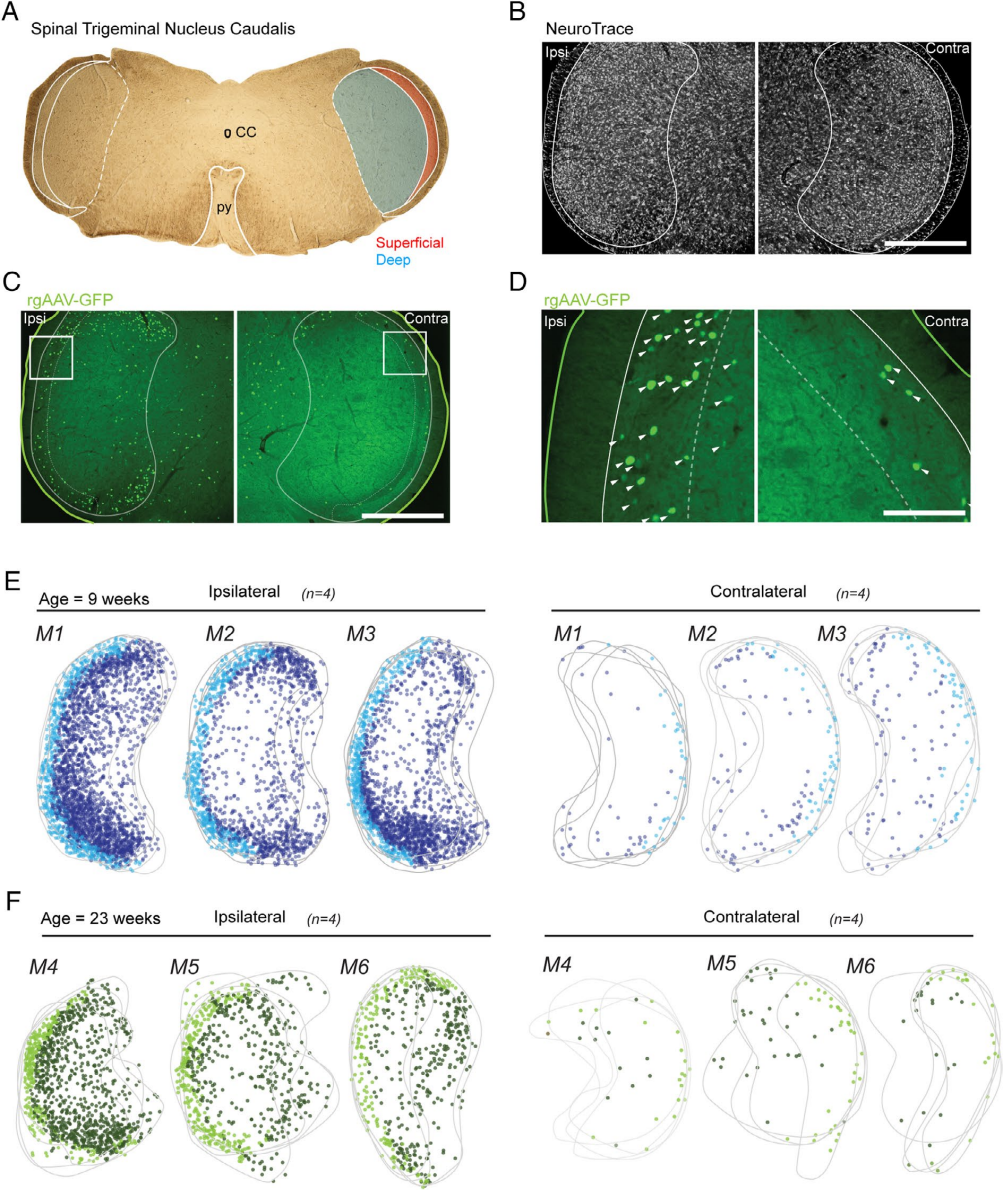
Retrograde transduction of Sp5C projection neurons by rgAAV‐GFP injection in young and older adult animals. (A) Brightfield image (4× objective) of labelled brainstem section (50 μm), showing key landmarks including the central canal (CC) and the pyramidal decussation (py), typical of sections included for analysis. Sp5C and the py are labelled with white outlines, with the Sp5C located laterally to the py. Sp5C was further classified into superficial (red) and deep (blue) regions that were differentiated for analysis also demarcated. (B–D). Fluorescent images show Sp5C labelling ipsilateral (Ipsi; left) and contralateral (Contra; right) to the PBN injection site. (B) Low magnification (10× objective) fluorescent image shows the ipsilateral and contralateral Sp5C regions labelled with NeuroTrace, identifying all neurons and helping differentiate the deep and superficial Sp5C based on neuronal density and packing. (C) Image of the same section as (B), showing rgAAV‐GFP labelling (green) used to identify retrogradely transduced Sp5C‐PBN projection neurons. The outline denotes the tissue section edge (solid; green line), with the Sp5C margin also labelled (solid; white line). The tissue is also further demarcated into deep and superficial Sp5C (dotted; white line). (D) High magnification (20x objective) images from (C), showing a typical nucleic labelling by rgAAV (white arrows). Strongest labelling was observed ipsilateral to the PBN injection. (E–F). Overlayed maps (grey) for M1, M2 and M3 (age: 9 weeks) and M4, M5 and M6 (age: 23 weeks) present all Sp5C‐PBN projection neuron distributions in ipsilateral and contralateral sections (*n* = 4 sections per animal). Marker colour denotes deep (dark blue/green), or superficial (light blue/green) Sp5C‐PBN projection neurons. Note the bias distribution, concentrated to the ipsilateral Sp5C. Scale: B, C: 500 μm, D: 100 μm.

In tissue prepared from rgAAV‐GFP injected animals, Sp5C‐PBN projection neurons were visualised within Sp5C (Figure 2A,B) as GFP labelling restricted to nuclei, associated with Cre recombinase expression (Figure 2C,D). This produced labelling across the entire extent of lamina I, both ipsilateral and contralateral to the PBN injection, with labelling also apparent in the dorsal and ventral terminal areas of lamina II (substantia gelatinosa). Comparisons of labelling across young adult animals (postnatal 9 weeks) showed some variability (Figure 2E; *n* = 3 animals, 4 sections per animal), with mouse one exhibiting more labelled Sp5C‐PBN projection neurons than mice two and three on the ipsilateral side (M1 vs. M2 vs. M3: 751 ± 170 vs. 407 ± 46 vs. 502.5 ± 73 neurons respectively; ANOVA: F(2, 9) = 10.369, *p* = 0.005; Tukey's post hoc: M1** > M2, M3, *p* = 0.004, *p* = 0.027 respectively), whereas Sp5C‐PBN projection neuron counts were not different in the contralateral Sp5C (M1 vs. M2 vs. M3: 40 ± 14 vs. 34 ± 8 vs. 18 ± 11 neurons; ANOVA: F(2, 9) = 4.166; *p* = 0.052). Given the similar relative distributions, these samples were also pooled across animals for a total sample of 6967 Sp5C‐PBN projection neurons, with > 94% of these located in the ipsilateral Sp5C (Ipsi vs. Contra: 6602 vs. 365 neurons respectively) and the average distribution per section differed significantly (Ipsi vs. Contra: 553 ± 181 vs. 30 ± 14 neurons, independent samples *t*‐test; *Z* = −9.959; *p* < 0.001). When the labelled Sp5C‐PBN projection neurons were classified by superficial or deep location, the deep population outnumbered the superficial cells, with a ratio of 1.75:1 in the ipsilateral Sp5C (Superficial vs. Deep; 201 ± 59 vs. 350 ± 151 neurons respectively; independent samples *t*‐test: *Z* = −3.183, *p* = 0.003), while there was no difference between the number of superficial and deep cells in the contralateral Sp5C (15 ± 10 vs. 15 ± 9 neurons; Mann–Whitney U test: *T* = 0.289, *p* = 0.799). This bias in distribution was also observed in the ipsilateral Sp5C when compared to the contralateral side in both regions (Ipsi vs. Contra: Superficial: 201 ± 59 vs. 15 ± 10 neurons; Mann–Whitney U test: *T* = 4.161, *p* < 0.001; Deep: 350 ± 151 vs. 15 ± 9 neurons; independent samples *t*‐test: *Z* = −7.642, *p* < 0.001). Labelling was also apparent in other regions in these sections, including areas surrounding the central canal, but was not formally analysed.

**FIGURE 3:**
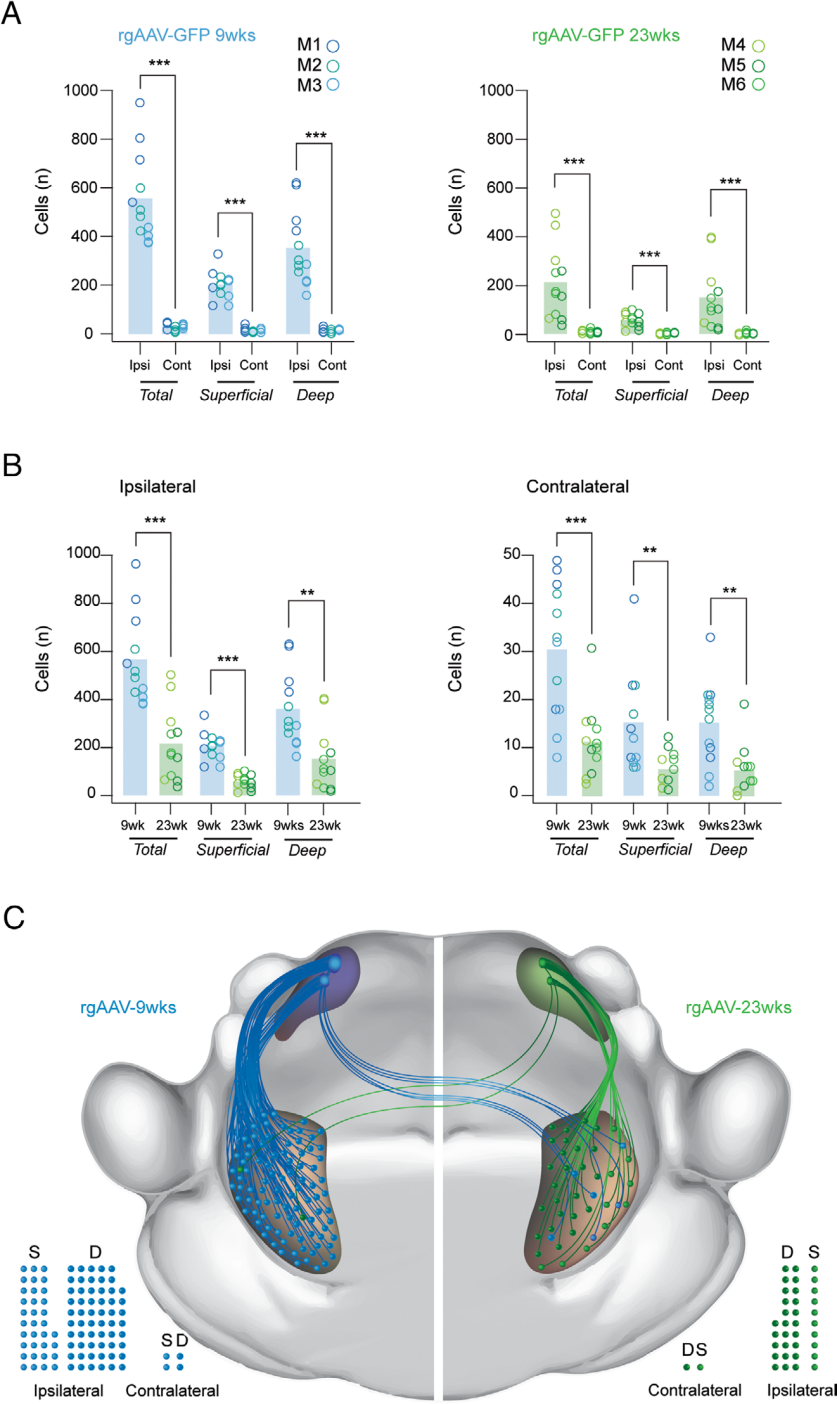
Age‐dependent labelling of Sp5C‐PBN projection neurons with rgAAV‐GFP in young and older adult mice. (A.) Group data plots compare rgAAV labelled Sp5C‐PBN projection neuron numbers ipsilateral and contralateral to the PBN injection site, in young (left) and older adult animals (right). Note the significant bias to ipsilateral labelling. (B) Group data plots compare rgAAV labelled Sp5C‐PBN projection neuron numbers in young and older adult mice in the ipsilateral (left) and contralateral Sp5C (right). Note significantly greater Sp5C‐PBN projection neurons are labelled when viral injections are performed in younger animals. (C) Schematic summarises the overall numbers and distribution of parabrachial projecting Sp5C projection neurons identified by rgAAV injection in young (right, blue) and older animals (left, green) in the superficial and deep Sp5C, both ipsilateral and contralateral to the viral injection site. Note that the ascending pathway represents the relative viral labelling efficacy and cell locations, but not the organisation of the specific fibre pathway connecting the Sp5C and the PBN, which was not characterised. Filled circles below also provide a comparison of Sp5C‐PBN projection neuron distributions in the superficial (S) and deep (D) regions of the ipsilateral and contralateral Sp5C. Statistics: **p < 0.01, ***p < 0.001.

To assess any impact of age on Sp5C‐PBN projection neuron labelling as well as the efficiency of Cre recombination, we also characterised labelling in older adult Ai32 animals (postnatal 23 weeks) that received rgAAV‐GFP injections (Figure 2F; *n* = 3 animals, 4 sections per animal). In these animals (M4, M5 and M6), there were fewer PNs captured on average across both the ipsilateral and contralateral Sp5C (Figure 3A,B; 23 weeks vs. 9 weeks: Ipsi: 211 ± 149 neurons vs. 550 ± 181 neurons; independent samples *t*‐test: *Z* = 4.979, *p* < 0.001; Contra: 12 ± 8 vs. 30.4 ± 14 neurons: independent samples *t*‐test: *Z* = 4.045; *p* < 0.001) which corresponded with a decreased total number of neurons (Total Ipsi: 2535 neurons; Total Contra: 140 neurons). Despite this observation, the ratio of ipsilateral: contralateral PN labelling was consistent with young adult tissue (> 94% within the ipsilateral Sp5C). Furthermore, there were decreased numbers across the superficial and deep regions in the ipsilateral (23 weeks vs. 9 weeks: Superficial: 60 ± 28 neurons vs. 201 ± 59 neurons; independent samples *t*‐test; *Z* = 7.44; *p* < 0.001; Deep: 151 ± 131 neurons vs. 350 ± 151 neurons; Mann Whitney U test: *T* = −3.0; *p* < 0.01) and contralateral Sp5C (23 weeks vs. 9 weeks: Superficial: 6 ± 4 neurons vs. 15 ± 10 neurons: Mann Whitney U test: *T* = −2.7; *p* < 0.01; Deep: 6 ± 5 neurons vs. 15 ± 9 neurons; Mann Whitney U test *T* = −2.8; *p* < 0.01). Despite these differences, the same relationship existed when comparing the ratio of Superficial to Deep PNs, which was 1:2.5 in the Ipsilateral Sp5C (Superficial/Deep: *n* = 725/1810) and 1.03:1 in the contralateral Sp5C (Superficial/Deep: *n* = 71/69). This highlights a deep bias of neurons within the ipsilateral Sp5C but similar numbers on the contralateral side (Figure 3C).

**FIGURE 4:**
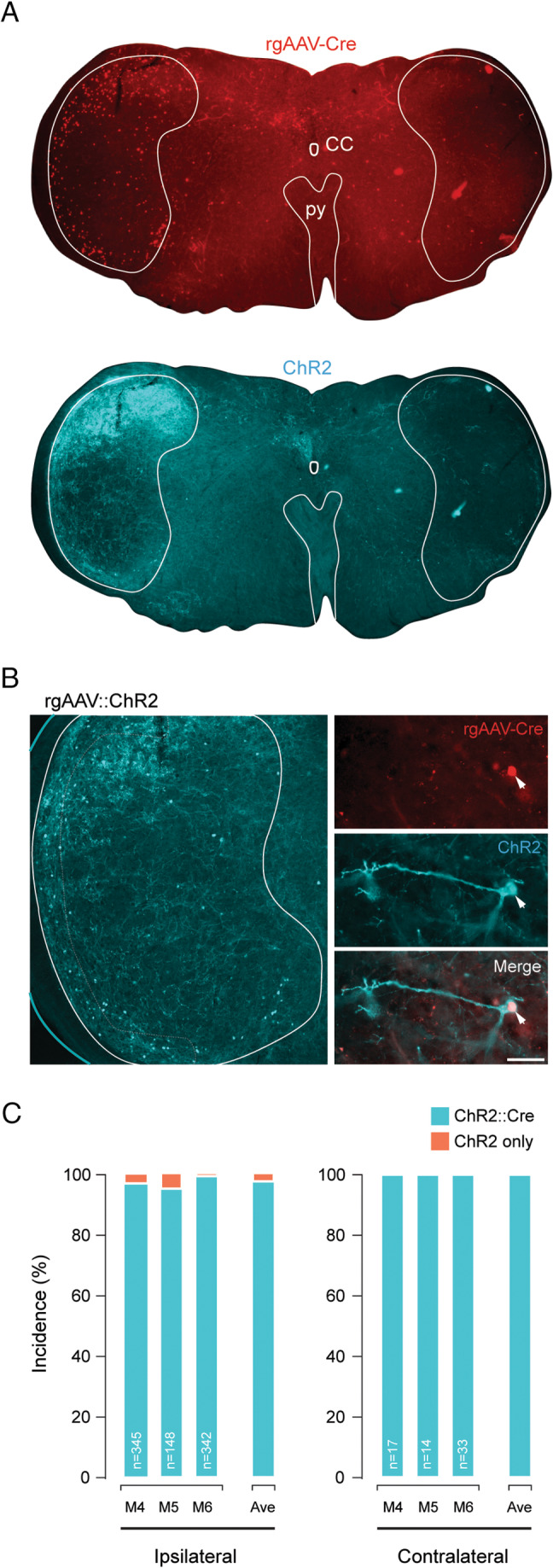
Cre‐dependent recombination mediated by rgAAV‐Cre injection in the parabrachial nucleus of transgenic Ai32 mice. (A) Low magnification (4× objective) images show a Sp5C containing brainstem section with Cre‐labelled Sp5C‐PBN projection neurons (upper, red) and Cre‐dependent ChR2 expression (lower cyan). Note the general overlap of labelling distribution and intensity for Cre and ChR2. (B) Single optical section of Sp5C (1 μm depth) highlighting the distinct labelling of cell bodies and processes in Sp5C‐PBN projection neurons (left). Higher magnification (40x objective) single optical section of an individual Sp5C‐PBN projection neuron showing rgAAV‐Cre expression (upper red), Cre‐dependent ChR2 expression (middle cyan) and merge (lower) highlighting overlap and successful Cre‐dependent recombination. (C) Plots summarise the reliability of Cre‐dependent ChR2 expression in the ipsilateral (left) and contralateral (right) Sp5C for individual Ai32 mice after rgAAV‐Cre injection of the left PBN. Number of ChR2‐expressing profiles assessed is presented on each bar and average (ave) across animals right of graphs. Note the high degree of recombination‐dependent profiles (i.e., 97.8% coexpressing Cre and ChR2) and very little ectopic expression (i.e., 2.2% expressing ChR2 without Cre).

Injection of rgAAV‐Cre in Ai32 animals (M4, M5 and M6) also allowed us to assess virally mediated Cre‐dependent recombination in Sp5C‐PBN projection neurons. This resulted in prominent ChR2 labelling observed in all sections that mirrored the distribution of Cre‐positive neurons (Figure 4A). The reliability of recombination‐dependent ChR2 expression was determined by assessing ChR2 Sp5C‐PBN projection neuron profiles for coexpression of Cre (Figure 4B). This showed a high degree of reliability with > 97% of ChR2 profiles also exhibiting Cre immunoreactivity in the ipsilateral Sp5C (Figure 4C, left; M4:339/345; M5:143/148; M6:340/342). Consistent with all previous analyses, there were fewer ChR2‐expressing projection neurons in the contralateral Sp5C; however, all coexpressed Cre (Figure 4C, right; M4:17/17; M5:14/14; M6:33/33). This confirms the utility of viral targeting strategies in the Sp5C of Cre‐dependent reporter lines.

### 3.2 AAV9 Labelling of Sp5C‐PBN Projection Neurons

**FIGURE 5:**
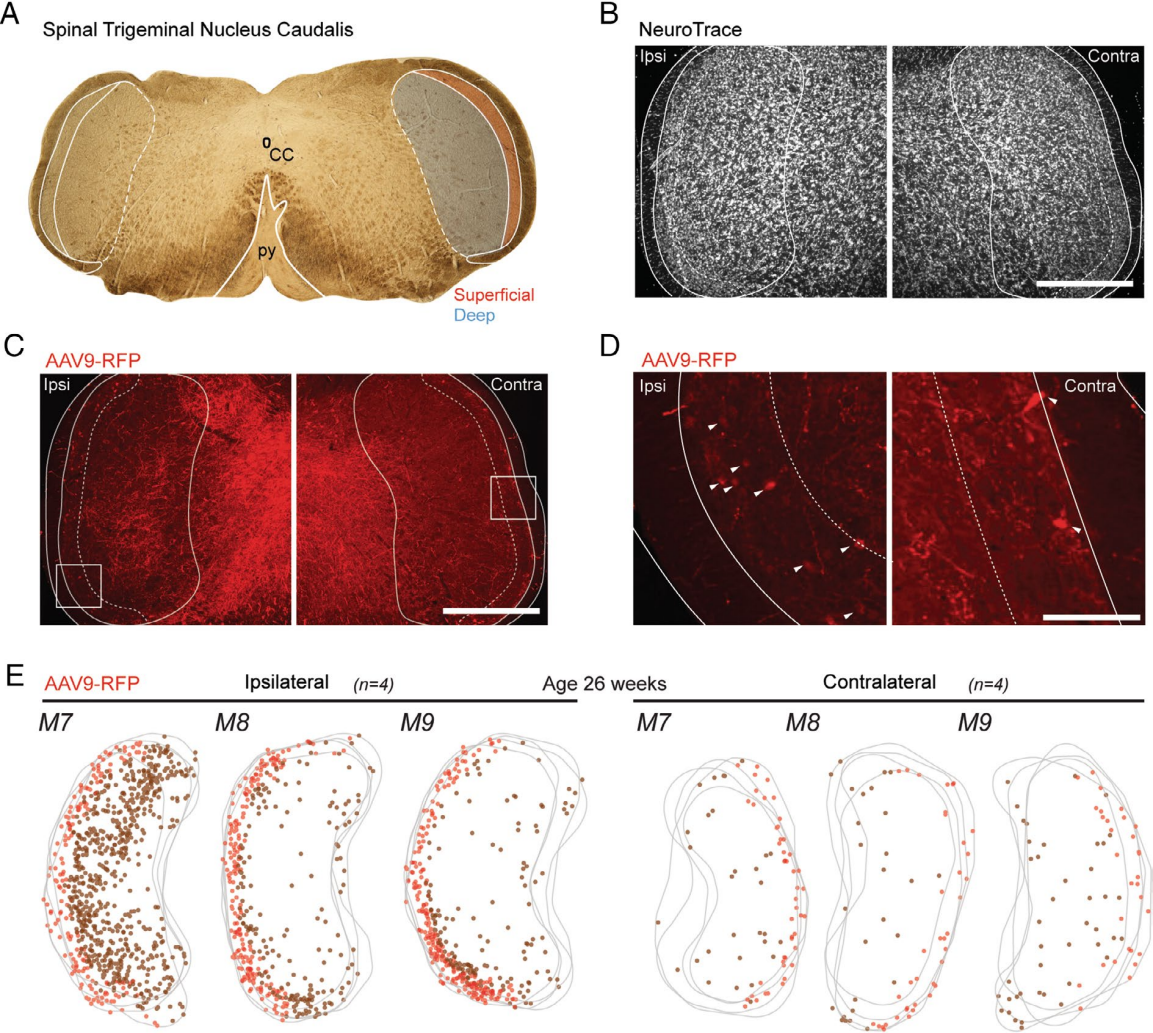
Retrograde transduction of Sp5C‐PBN projection neurons by AAV9‐RFP injection in older adult mice. (A). Brightfield image (4× objective) of labelled brainstem section (50 μm) showing key landmarks, including the central canal (CC) and the pyramidal decussation (py) with the Sp5C located laterally to the py. Sp5C is shown with white outlines, with superficial (red) and deep (blue) Sp5C regions that were differentiated for analysis also demarcated. (B–D). Fluorescent images (10× objective) show Sp5C labelling ipsilateral (Ipsi) and Contralateral (Contra) to the PBN injection. (B). Shows the ipsilateral and contralateral Sp5C regions labelled with NeuroTrace, identifying all neurons and helping differentiate the deep and superficial Sp5C, based on neuronal density and packing. (C). Image of same sections as (B), showing AAV9‐RFP labelling (red) used to identify retrogradely transduced Sp5C‐PBN projection neurons. The outline denotes the tissue section edge (solid; white line), with the Sp5C margin also labelled (solid; white line). The tissue is also further demarcated into deep and superficial Sp5C (dotted; white line). (D). High magnification (20× objective) image from C showing AAV9‐RFP labelling (white arrows). (E). Overlayed maps of Sp5C (grey) for M7, M8 and M9 present AAV9‐RFP (*n* = 4 sections per animal), labelled Sp5C‐PBN projection neuron distributions in the ipsilateral (left) and contralateral (right) Sp5C. Marker colour denotes deep (brown), or superficial (red) Sp5C‐PBN projection neurons. (C–E). Note that the bias distribution concentrated to the ipsilateral Sp5C. Scale: B, C: 500 μm, D: 100 μm.

AAV9‐RFP injected animals displayed a similar distribution of transduced Sp5C‐PBN projection neurons within Sp5C (Figure 5A,B) to the rgAAV‐Cre tissue; however, this construct labelled somatic, dendritic and axonal profiles (Figure 5C,D), rather than the nuclear restricted in rgAAV‐Cre injections. Thus, a range of ascending and descending fibre tracts arising from the PBN injection zone were also labelled. At the level of Sp5C, this axonal labelling was prominent in the deeper laminae ipsilateral to the PBN injection site, but less so within the contralateral side. There was variability in the neuronal labelling across animals (Figure 5E; *n* = 3 animals, 4 sections each) with mouse one (M7) having more Sp5C‐PBN projection neurons per section than the remaining two mice (M8 and M9) on the ipsilateral side (M7 vs. M8 vs. M9: 210 ± 66 vs. 100 ± 18 vs. 102 ± 19 respectively; ANOVA: F(2, 9) = 9.228; *p* = 0.007; Tukey's post hoc: M7** > M8, M9, *p* = 0.011, *p* = 0.013 respectively) but as with the rgAAV‐GFP results, there was no difference on the contralateral side (M7 vs. M8 vs. M9: 18 ± 5 vs. 16 ± 6 vs. 19 ± 7 neurons respectively, ANOVA; F(2, 9) = 0.287, *p* = 0.757). Regardless, when results from animals were pooled, there was a total of 1865 Sp5C‐PBN projection neurons labelled across the sections, with 88% of all profiles found in the ipsilateral Sp5C (Ipsi vs. Contra; 1649 vs. 216 Sp5C‐PBN projection neurons). When compared for individual section totals, this average distribution was statistically different (Ipsi vs. Contra: 137 ± 65 vs. 18 ± 5 neurons; Mann–Whitney U test: *T* = 4.158; *p* < 0.001). The data were also grouped into superficial and deep populations for comparison, but there was no difference in the number of superficial versus deep Sp5C‐PBN projection neurons labelled in the ipsilateral (Superficial vs. Deep: 60 ± 14 vs. 77 ± 67 neurons; Mann–Whitney U test: *T* = −1.271; *p* = 0.219) or contralateral (Superficial vs. Deep: 10 ± 5 vs. 8 ± 5 neurons, Mann–Whitney U test: *T* = −1.422; *p* = 0.16) Sp5C regions. There were, however, significantly more superficial and deep neurons in the Sp5C region ipsilateral to PBN injection when compared to the corresponding contralateral Sp5C (Ipsi vs. Contra: Superficial: 60 ± 15 vs. 10 ± 5 neurons; independent samples *t*‐test: *T* = −11.32, *p* < 0.001; Deep: 77 ± 67 vs. 8 ± 5 neurons; Mann–Whitney U test: *T* = 4.162; *p* < 0.001).

### 3.3 Comparison of AAV9 and rgAAV Labelling in Sp5C‐PBN Projection Neurons

**FIGURE 6:**
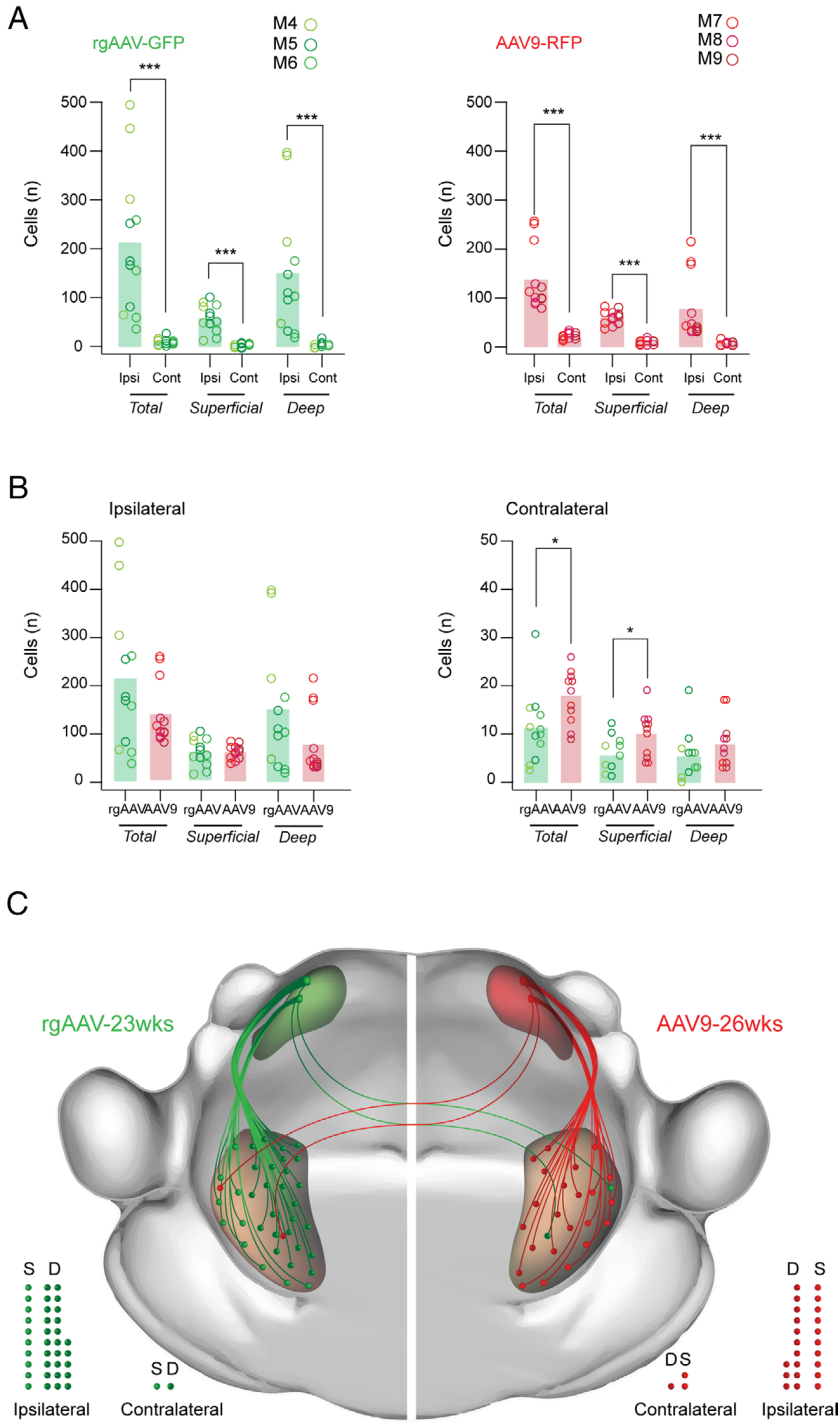
Comparison of Sp5C‐PBN projection neuron labelling with rgAAV‐GFP and AAV9 viruses. (A). Group data plots present rgAAV‐GFP (left; *n* = 3; M4, M5, M6 age: 23 weeks) and AAV9‐RFP (right; *n* = 3; M7, M8, M9 age: 26 weeks) labelled Sp5C‐PBN projection neuron numbers from Figures 3 and 5 across Sp5C. Note that significantly greater cell numbers were identified within the ipsilateral Sp5C compared to the contralateral side for both vectors. This bias distribution was also true when Sp5C‐PBN projection neurons were separated into superficial and deep Sp5C locations. (B). Group data plots comparing rgAAV‐GFP (green) and AAV9‐RFP (red) labelled Sp5C‐PBN projection neuron numbers within the ipsilateral (left) and contralateral (right) Sp5C. Both vectors labelled similar cell numbers on the ipsilateral Sp5C, however, AAV9‐RFP experiments labelled more Sp5C‐PBN projection neurons in the contralateral Sp5C. These relative distributions were also largely preserved when cells were categorised as superficial or deep, except for the deep contralateral population where numbers were the number of labelled cells was similar between rgAAV‐GFP and AAV9‐RFP. (C). Schematic summarises the overall numbers and distribution of parabrachial projecting Sp5C neurons identified by rgAAV (left, green) and AAV9 (right, red) in the superficial and deep Sp5C, both ipsilateral and contralateral to the viral injection site. Note that the ascending pathway represents the relative viral labelling efficacy and cell locations, but not the organisation of the specific fibre pathway connecting the Sp5C and the PBN, which was not characterised. Filled circles below also provide a comparison of Sp5C‐PBN projection neuron distributions in the superficial (S) and deep (D) regions of the ipsilateral and contralateral Sp5C. Statistics: *p < 0.05, ***p < 0.001.

Finally, Sp5C‐PBN projection neuron labelling numbers and distributions achieved by rgAAV‐GFP and AAV9‐RFP injections were compared in age‐matched animals (M4, M5, M6 vs. M7, M8, M9) to assess their relative utility for future research (Figure 6A; *n* = 3 animals, 4 sections per animal). Consistent with all previous comparisons, the greatest number of Sp5C‐PBN projection neurons was on the ipsilateral Sp5C and these numbers were similar between the rgAAV‐GFP and AAV9‐RFP tissue (Figure 6B,C: 211 ± 149 neurons vs. 137 ± 65 neurons: Mann–Whitney U test: *T* = 0.87; *p* > 0.4). This similarity remained when counts were separated into superficial and deep Sp5C‐PBN projection neurons (ipsi superficial rgAAV; AAV9: 60 ± 15 neurons vs. 60 ± 28 neurons; independent samples *t*‐test: *Z* = 0.00; *p* > 0.99; ipsi deep rgAAV; AAV9: 150 ± 130 neurons vs. 77 ± 70 neurons; Mann–Whitney U test: *T* = 1.36; *p* > 0.15). In contrast, there were differences in Sp5C‐PBN projection neuron numbers in the contralateral Sp5C, with AAV9 injection yielding more labelled profiles (rgAAV vs. AAV9: 12 ± 8 neurons vs. 18 ± 5 neurons: Mann–Whitney U test: *T* = −2.34; *p* < 0.05). This difference remained in the superficial Sp5C when Sp5C‐PBN projection neuron counts were segregated by region (contra superficial rgAAV; AAV9: 6 ± 4 neurons vs. 10 ± 5 neurons: independent samples t‐test: *Z* = 2.5; *p* < 0.05), but labelling was similar in the contralateral deep Sp5C (contra deep rgAAV; AAV9: 6 ± 5 neurons vs. 8 ± 5 neurons; Mann–Whitney U test: *T* = −1.28; *p* > 0.2).

## 4 Discussion

This study sought to assess the efficacy of two different viral constructs injected into the PBN of mice to label and identify projection neurons in the Sp5C. We found that both viruses produced substantial labelling in the superficial and deep Sp5C ipsilateral to the PBN injection. Sp5C‐PBN projection neurons were also captured in the contralateral Sp5c, though at consistently lower levels. When the impact of animal age on relative labelling was compared, we showed that injection in younger animals produced more efficient transduction and labelling. Comparison between viral constructs suggested both rgAAV and AAV9 broadly identified similar Sp5C‐PBN projection neuron numbers, apart from a small difference in the contralateral Sp5C (Figure 5E). Finally, we confirmed successful Cre‐dependent expression can achieve efficient ChR2 expression in Sp5C‐PBN projection neurons, opening up many future experimental manipulations to study this population. The following discussion highlights important technical considerations, comparisons with previous findings and the functional relevance of these results.

### 4.1 Technical Considerations

There are several important considerations that require consideration for interpreting these findings. For example, it is well established that projection neurons in the spinal somatosensory pathway give rise to axons that predominantly cross the midline and ascend in the contralateral white matter (Chen et al. 2024). While literature suggests that this organisation is less distinct in the brainstem, we were surprised to observe such large numbers of Sp5C‐PBN projection neurons in the ipsilateral versus contralateral Sp5C, with up to 20 times more neurons ipsilaterally labelled. This is consistent with other findings (Saito et al. 2017; Wang et al. 2001), but in stark contrast to spinoparabrachial projection neurons. Another clear difference was in the age effect we observed using rgAAV in young and older adult mice, with rgAAV producing greater Sp5C‐PBN projection neuron labelling in younger animals. It is well documented that viral labelling can exhibit age dependence in some CNS regions (Polinski et al. 2016; Sandoval et al. 2024), so one conclusion from this finding is that age should be a consideration for future Sp5C‐PN studies employing AAV targeting strategies. Furthermore, we were not able to comment on sexual dimorphism as a potential factor in the ascending somatosensory system, and this will need to be the focus of future studies.

Another consideration for our data is the nature of the labelling using different constructs, with AAV9‐mCherry virus producing high levels of terminal labelling throughout the Sp5C, as well as in the cell bodies and dendrites. This could be observed as prominent fluorescence throughout the MDH sections (Figure 4C) and could have obscured faint somatic labelling, leaving such profiles undetected and excluded from our analysis. By comparison, the rgAAV construct unambiguously labelled Sp5C‐PBN projection neuron nuclei (Figure 3C) and allowed for easier identification and quantification. Our use of confocal imaging, assessment across multiple optical sections and clear inclusion criteria suggest that this difference would not substantially contribute to the reported numbers. Relating to the location, our analysis also did not distinguish specific laminae within the Sp5C (as in Gobel et al. 1977); rather, we separated Sp5C‐PBN projection neurons by location as either superficial or deep Sp5C. This follows the well‐characterised arrangement of spinoparabrachial projection neurons distributed in LI, LII and LV and rarely in other laminae. Therefore, we can be confident that Sp5C‐PBN projection neurons in the superficial Sp5C included laminae I and II, and the deep Sp5C‐PBN projection neurons were located in lamina IV and V, but do not assign cells to specific laminae.

The particularly high number of Sp5C‐PBN projection neurons was a somewhat surprising finding, contrasting with the lumbar spinal cord, where we have reported lower overall numbers and a contralateral dominance in the superficial DH, but equivalent projection neuron numbers in the ipsilateral and contralateral deep DH (Browne et al. 2024). Importantly, both studies used the same injection technique, viral incubation times and the same AAV9‐RFP virus. The larger number of projection neurons labelled here in the brainstem could reflect the proximity of the virus injection (i.e., PBN) to the region of analysis, that is, the mouse Sp5C, versus the lumbar spinal DH. This observation may also reflect the importance in ensuring that potentially damaging stimuli to the face are faithfully relayed and perceived. This result may also be related to the functionally complex, homeostatic PBN receiving dense innervation from the brainstem, whose functional connectivity remains poorly understood. The nature of viral injections also has the potential to impact labelling, with the possibility of viral spread into neighbouring nuclei that may receive input from Sp5C, such as the periaqueductal grey, inferior colliculi, pontine tegmental areas and locus coeruleus and so on. The outcome of our injection site analysis, however, was typical of other studies targeting the PBN including our previous work (Browne et al. 2024; Browne et al. 2021; Cameron et al. 2015); therefore, this result most likely indicates a complexity to the pathways that interconnect the Sp5C and PBN.

### 4.2 Comparison With Previous Work

The PBN is an extremely complex nucleus, with over 20 subclusters of neurons that contribute to the response to noxious stimuli, food and water intake, sleep regulation, respiration and fear conditioning, all requiring a wide range of afferent inputs (Pauli et al. 2022). Among these inputs, consistent targeting of PBN by ascending projection neurons of the spinal cord and medulla is widely reported (Feil and Herbert 1995). Although the nuances of the trigemino‐parabrachial pathway are not yet fully appreciated, studies consistently describe that most Sp5C PNs connect with the ipsilateral PBN and target the Kölliker–Fuse and external lateral subdivisions of the PBN. For instance, fluorogold tracing of Sp5C‐PBN projections (Akiyama et al. 2016; Tokita et al. 2009) produced a labelling pattern similar to our report, with the highest density of Sp5C‐PBN projection neurons in the superficial region of the ipsilateral brainstem. However, we also identify a population of deep Sp5C‐PBN projection neurons labelled by both viral constructs (Figure 5B). This is hard to reconcile due to the lipophilic nature of classic tracers with potential uptake by axons of passage that can impact labelling yields and distort termination zones. One potential factor in this difference is the often particulate nature of classic tracer labelling that may lead to an underprediction of numbers, whereas viral constructs typically produce clear labelling of the entire neuron, or nucleus, when transfected. Although collectively the current data report greater numbers, it appears that the ratio of contralateral to ipsilateral Sp5C‐PBN projection neurons is similar using these methods (Figure 5E). Interestingly, previous work has shown distinct uneven Sp5C‐PBN projection neuron distributions throughout the dorsal/dorsolateral region of Sp5C, and within both superficial and deep regions (Nakaya, Yamamoto, and Kobayashi 2023). This study injected cholera toxin subunit B (CTB) conjugated with Alexa Fluor 647 into the PBN and an AAV‐ChR2‐mCherry into the inferior colliculus of rats. The result was relatively low numbers of Sp5C‐PBN projection neurons compared with the current data, concentrated within the dorsal to dorsolateral Sp5C. The absence of a similar bias in the current data may be influenced by the viral tracing strategy, as well as a species difference, or a difference in the nature of injections.

Several studies have also used the viral tracing approach employed here to assess spinoparabrachial projection neurons located in the Dorsal Horn (Bell et al. 2024; Browne et al. 2021; Chen et al. 2024; Ford, Ren, and Baccei 2018). These listed studies have utilised a range of vectors including AAV1, AAV9, rgAAV‐GFP and rgAAV‐CAG‐tdTomato, and in contrast to Sp5C‐PBN projecting neurons, the majority of spinoparabrachial neurons are routinely reported in the DH contralateral to the PBN injection (Cameron et al. 2015). For example, labelling of spinoparabrachial projection neurons with AAV9 has been shown to yield cells concentrated in lamina I of the contralateral DH, with more scarce populations observed in lamina III/IV on both the ipsilateral and contralateral sides of the DH. This contrasts with the current AAV9 labelling of Sp5C‐PBN projection neurons, with dominant ipsilateral labelling and comparable numbers observed in the superficial and deep Sp5C (Figure 4A). Labelling with rgAAV‐hSyn/CAG constructs offers an exception to this general pattern, with equivalent spinoparabrachial projection neuron labelling in the ipsilateral and contralateral DH concentrated in LV of the deep DH (Bell et al. 2024; Browne et al. 2024). In addition, rgAAV‐hSyn/CAG labelled a much lower density of spinoparabrachial projection neurons than our observations for the Sp5C‐PBN population using the same serotype and promoter. Specifically, our data show an average of 2x more contralateral Sp5C‐PBN projection neurons, and up to 70x more ipsilateral Sp5C‐PBN projection neurons, compared to numbers reported in the spinal cord (Browne et al. 2021). Finally, a direct comparison of spinoparabrachial projection neuron labelling with AAV9‐RFP and rgAAV‐ChR2/GFP viral vectors showed that AAV9‐RFP PBN injections yielded a population of spinoparabrachial projection neurons 10× higher than that identified for rgAAV‐ChR2/GFP PBN injections (Browne et al. 2024). In contrast, the current results identified similar numbers of Sp5C‐PBN projection neurons labelled with rgAAV and AAV9 viruses. Together, these comparisons highlight distinct transduction patterns for viral serotypes even when employed in similar neuronal pathways and circuits (i.e., spinoparabrachial versus trigemino‐parabrachial.) These distinct results are likely influenced by differences in ascending sensory pathways responsible for relaying noxious information from the body (spinal DH) and ascending orofacial (Sp5C) regions to the PBN.

### 4.3 Functional Relevance and Future Direction

This study demonstrates the utility of AAVs to study trigemino‐parabrachial projection neurons, consistent with previous work studying spinoparabrachial projection pathways (Bell et al. 2024; Browne et al. 2021; Chen et al. 2024; Choi et al. 2020). High levels of transduction in Sp5C‐PBN projection neurons for both AAV9‐RFP/Cre and rgAAV‐GFP indicate that they are suitable vectors for studying the links between the trigeminal nucleus caudalis and the parabrachial nucleus. Future work using dual AAV9/rgAAV injections in the PBN and subsequent double labelling analysis will be required to assess the degree of overlap in expression produced by both vectors. This will be relevant as an expanding literature on the spinal cord suggests that subpopulations of projection neurons exist which mediate distinct aspects of sensory experiences (Chen et al. 2024; Choi et al. 2020; Hachisuka, Koerber, and Ross 2020). The presence of nonoverlapping AAV9 and rgAAV Sp5C projection neuron populations would add evidence for a similar level of organisation in Sp5C. This may allow for the utilisation of tropism to understand different elements of the pathways that relay trigeminal nerve inputs to higher brain regions.

To our knowledge, adeno‐associated viruses are yet to be widely used in Sp5C‐PBN projection neuron studies (Title et al. 2024) and the current results provide valuable information to selectively study these pathways. The use of viruses in place of classic tracers provides the opportunity to achieve superior labelling of cell bodies, axons and dendritic processes of projection neurons, allowing future studies to undertake detailed morphological analyses. In addition, the validation of Cre‐expressing AAVs to transduce projection neurons in Sp5C opens the opportunity to use intersectional viral strategies utilising Cre‐dependent viruses (as in (Browne et al. 2024; Chen et al. 2024), transsynaptic viruses, as well as recombination in Cre‐dependent animal lines to study the function of Sp5C‐PBN projection neurons using calcium imaging (Yarmolinsky et al. 2024) and optogenetics (Stemkowski et al. 2016) or chemogenetic actuators/inhibitors (Nakaya, Yamamoto, and Kobayashi 2023). Intersectional strategies will also be helpful to better understand the varied outputs from the Sp5C to other midbrain and brainstem regions beyond the PBN. For example, trigemino‐thalamic projection neurons have previously been targeted using retrograde fluorogold labelling, and this work could be further enhanced by a viral approach (Akiyama et al. 2016). Finally, while this work has validated two AAV serotypes to study Sp5C‐PBN projection neurons, future studies of additional AAV subtypes in this region should continue to establish alternative projection neuron labelling strategies that could be advantageous for future experiments (Aschauer, Kreuz, and Rumpel 2013; Haenraets et al. 2017). In summary, establishing the utility and selectivity of viral vectors to label projection neuron populations in the Sp5C is an important step in implementing genetic approaches in neuroscience that will facilitate new experimental paradigms to better understand the circuit‐based origins of orofacial pain in health and disease.

## Author Contributions

**Sophie Maric:** writing – original draft, investigation, writing – review and editing, formal analysis, visualization. **Mehedi Hasan:** formal analysis, writing – review and editing. **Madison L. Pounder:** writing – review and editing, formal analysis. **Brett A. Graham:** conceptualization, funding acquisition, writing – original draft, writing – review and editing, supervision, methodology, project administration, investigation, resources, visualization. **Tyler J. Browne:** conceptualization, investigation, writing – original draft, methodology, validation, writing – review and editing, supervision, data curation, project administration, funding acquisition.

## Conflicts of Interest

The authors declare no conflicts of interest.

### Peer Review

The peer review history for this article is available at https://www.webofscience.com/api/gateway/wos/peer‐review/10.1111/jnc.70028.

## Data Availability

Data will be made available upon reasonable request.
